# Volume XXX.—Omega

**Published:** 1891-07

**Authors:** 


BUFFALO MEDICAL AND SURGICAL JOURNAL

A MONTHLY REVIEW OF MEDICINE AND SURGERY.

*EDITORS:*

THOMAS LOTHROP, M. D. - - WM. WARREN POTTER, M. D.

All communications, whether of a literary or business character, should be addressed to the editors : 284 FRANKLIN STREET, BUFFALO, N. Y.

VOL. XXX. JULY, 1891. No. 12.

### Editorial.

### VOLUME XXX.—OMEGA.

WITH this number, the thirtieth volume of this magazine reaches its end. We hope our readers are satisfied with the results that the year has attained. They certainly have cost us much labor, and if our patrons accord us their approbation, we shall feel fully and amply compensated for it all. Our Associate Staff has been of valuable assistance to us in the work of the year, and we here and now express to them, each and all, our cordial thanks.

The index which accompanies the number will be found a valuable reference list, and especially convenient for those who bind the JOURNAL.

